# Effects of Clay Therapy on Emotional and Physical Outcomes in Hospitalized Pediatric Cancer Patients: A Prospective Pilot Study

**DOI:** 10.3390/cancers18071128

**Published:** 2026-04-01

**Authors:** Antonella Guido, Alberto Romano, Laura Peruzzi, Matilde Tibuzzi, Serena Sannino, Paola Adamo, Daniela Pia Rosaria Chieffo, Antonio Ruggiero

**Affiliations:** 1 Unità Operativa Complessa Oncologia Pediatrica, Dipartimento della Salute della Donna e del Bambino, Fondazione Policlinico Universitario Agostino Gemelli IRCCS, 00167 Rome, Italy; antonella.guido@guest.policlinicogemelli.it (A.G.); laura.peruzzi@guest.policlinicogemelli.it (L.P.); serena.sannino@guest.policlinicogemelli.it (S.S.);; 2 Unità Operativa Semplice Psicologia Clinica, Fondazione Policlinico Universitario Agostino Gemelli IRCCS, 00167 Rome, Italy; 3 Fondazione Lene Thun, 39100 Bolzano, Italy; matilde-t@hotmail.com (M.T.);; 4 Dipartimento della Salute della Donna e del Bambino, Università Cattolica del Sacro Cuore, 00168 Rome, Italy

**Keywords:** pediatric cancer, clay therapy, creative arts therapy, supportive care, quality of life, emotional well-being, psychosocial intervention, hospitalization

## Abstract

**Background**: Pediatric cancer is a highly stressful and potentially traumatic condition affecting physical health, emotional well-being, developmental trajectories, and family functioning. Hospitalization and intensive treatments often exacerbate emotional distress and symptom burden, negatively impacting quality of life (QoL). Integrating psychosocial and expressive interventions into pediatric oncology care is increasingly recognized as essential. Clay therapy is a multisensory, hands-on creative intervention that may promote emotional regulation, coping, and a sense of agency in hospitalized children. **Objectives**: This prospective pilot study evaluated the effects of clay therapy on emotional and physical well-being in pediatric oncology patients and explored potential indirect effects on caregivers’ emotional status. **Methods**: From December 2023 to December 2024, forty hospitalized children with onco-hematological diseases and one parent per patient were enrolled. Each child participated in a one-hour clay therapy workshop led by a professional ceramist. Emotional outcomes were assessed before (T0) and immediately after (T1) the intervention using the Visual Analog Scale (VAS) and the ArtsObs observational scale. Physical symptoms, including pain, fatigue, and nausea, were also evaluated. **Results**: Following clay therapy, children showed statistically significant improvements across all VAS emotional domains, independent of age and gender. ArtsObs assessments confirmed a significant increase in observed mood, with high levels of relaxation and engagement; female patients demonstrated greater mood improvement than males. Significant reductions in pain, fatigue, and nausea were also observed. Parents exhibited a significant improvement in mood following their child’s participation. **Conclusions**: These findings suggest that clay therapy is feasible and could be an effective supportive intervention in pediatric oncology, benefiting both emotional and physical well-being. Its integration into multidisciplinary, family-centered care models may enhance QoL during hospitalization and provide holistic support for children with cancer and their families.

## Simple Summary

Children hospitalized for cancer often experience emotional distress, physical discomfort, and reduced quality of life. Supportive interventions addressing psychological and developmental needs may help alleviate these difficulties alongside medical treatment. This pilot study investigated whether clay therapy, a structured creative activity involving hands-on clay modeling, could improve emotional and physical well-being in children with cancer and indirectly benefit their parents. Forty pediatric patients with onco-hematological diseases participated in a one-hour clay therapy session during hospitalization. Emotional state, pain, fatigue, and nausea were assessed before and after the activity, and parents’ emotional well-being was also evaluated. After clay therapy, children showed significant improvements in mood and emotional regulation, together with reductions in anxiety, anger, pain, fatigue, and nausea. Observational measures confirmed increased relaxation and engagement during the sessions. Parents also experienced a significant improvement in mood following their child’s participation. These findings indicate that clay therapy is a feasible, low-risk, and potentially valuable supportive intervention that may enhance quality of life for children with cancer and support family well-being during hospitalization.

## 1. Introduction

Pediatric cancer represents a profoundly stressful and potentially traumatic life event due to both the severity of the disease and the complexity of its treatments. Childhood and adolescence are periods of rapid and dynamic development, during which biological, cognitive, emotional, and social systems are interdependent and highly sensitive to environmental influences. The diagnosis of a neoplastic disease constitutes a life-altering event [1], imposing significant strain on the physical, emotional, and existential development of children and adolescents. Beyond the immediate physiological impact, cancer introduces multiple challenges, including pain, fatigue, functional limitations, hospitalization-related disruption, and existential concerns [2,3,4]. These factors may profoundly influence self-perception, body image, identity formation, and emerging autonomy [5].

The psychosocial and developmental implications of cancer and its treatment have been widely documented. Children with cancer often experience heightened fear, anxiety, distress, and social isolation, which can compromise both adaptation to illness and everyday functioning [6,7,8,9,10,11]. These experiences may disrupt normative developmental trajectories, affecting cognitive, emotional, and relational growth. Families, and particularly primary caregivers, are also significantly affected, showing increased risk of emotional exhaustion, anxiety, and depressive symptoms due to the chronic and life-threatening nature of pediatric cancer. Recognizing these psychosocial challenges, international and national guidelines emphasize that psychological interventions should be integrated within a family-centered, multidisciplinary care model, addressing medical, psychological, and social needs concurrently [12].

Hospitalization represents a critical developmental and emotional challenge. The sudden transition from a familiar home environment into a highly medicalized and unpredictable hospital context constitutes a non-normative developmental crisis [13,14]. Children must adapt to institutional routines, cope with separation from family, endure procedural pain, and confront loss of autonomy and privacy. These stressors may manifest as emotional and behavioral disturbances, including elevated anxiety and depression, reduced self-esteem, and reliance on predominantly avoidant or repressive coping strategies [15,16,17,18]. Longitudinal studies suggest that while distress peaks immediately after diagnosis, children and families gradually activate adaptive strategies, reestablishing routines that foster resilience and mitigate uncertainty [19,20].

Within pediatric oncology, the concept of quality of life (QoL) has become a central outcome, extending beyond mere survival to include physical, emotional, cognitive, and social domains [21,22]. QoL evaluation provides a comprehensive understanding of the child’s experience of illness and treatment, informing interventions that support psychosocial adaptation, developmental progression, and emotional well-being. Assessment of QoL also highlights the importance of addressing secondary outcomes, such as coping strategies, self-efficacy, and resilience, which are critical in shaping long-term psychological adjustment.

Expressive and creative interventions, including play-based activities, art therapy, music therapy, and drama, have been increasingly recognized as essential components of supportive care in pediatric oncology [23,24,25]. These interventions provide distraction from medical procedures, foster engagement, enable emotional expression, and promote coping and self-efficacy. Engagement in creative activities may also influence physiological processes, including reduction in cortisol levels, modulation of autonomic nervous system activity, and enhanced reward-related neural activation, contributing to overall stress reduction [26,27].

Among expressive modalities, clay therapy has emerged as a particularly promising intervention. Clay therapy involves structured, hands-on creative activities where children manipulate, model, and color clay, enabling multisensory stimulation, emotional expression, and cognitive engagement. The tactile and kinesthetic aspects of clay manipulation engage both somatosensory and proprioceptive systems, promoting grounding and emotional regulation [28,29]. For hospitalized children, whose opportunities for autonomous play and social interaction are limited, clay therapy provides a unique avenue for agency, self-expression, and mastery. The process of shaping clay can symbolically represent internal experiences, allowing children to externalize complex feelings such as fear, frustration, or hope, which might otherwise remain unarticulated.

Although art-based interventions have been shown to reduce anxiety, improve mood, and enhance coping in pediatric patients [30,31,32], empirical evidence specifically examining clay therapy in pediatric oncology remains scarce. Preliminary observations suggest that clay therapy may improve mood, reduce stress and anger, enhance arousal regulation, and foster a sense of control and self-efficacy. Additionally, these interventions may indirectly benefit caregivers by alleviating their perceived emotional burden and enhancing the overall family coping environment [33].

Based on this theoretical and empirical background, the present pilot study aimed to evaluate the effects of clay therapy on emotional and physical well-being in hospitalized pediatric oncology patients, as well as potential indirect benefits on caregivers. By integrating clay therapy into a multidisciplinary supportive care model, this study emphasizes the importance of addressing not only the physical dimensions of disease but also the psychosocial, developmental, and emotional needs of children with cancer, supporting holistic care and resilience in the face of life-threatening illness.

## 2. Materials and Methods

### 2.1. Study Endpoints

The primary endpoint was:

- to evaluate the effect of clay therapy on emotional and physical status of children with onco-hematological disease.

The secondary endpoints were:

- to evaluate the effect of clay therapy on emotional status of the parents;
- to evaluate the relationship between the demographic characteristics of the children and of the parents and the effectiveness of clay therapy on emotional status.

### 2.2. Study Design, Population and Ethical Approval

From December 2023 to December 2024, we conducted a prospective pilot study enrolling patients with pediatric onco-hematological disease admitted to the Pediatric Oncology Unit of Fondazione Policlinico Agostino Gemelli IRCCS of Rome. The study involved also the parents of the patients.

Inclusion criteria were diagnosis of oncological disease, age over 5 years, regular hospitalization for chemotherapy, absence of acute complications (infections, mucositis, other concomitant diseases).

Exclusion criteria were age under 5, emergent hospitalization required for complications related to the underlying disease or treatment, visual or hearing difficulties that prevent the complete collection of information required by the tests.

Each patient was offered a clay therapy session, which consisted of a ceramics workshop led by an experienced master ceramist in the presence of the department’s psychologist, during which the patient could directly model and color the clay. Each workshop lasted approximately one hour. Each session involved small groups of 3–4 patients, homogeneous in age in order to foster an appropriate relational climate and balanced participation. The clay therapy session were conducted jointly by a master ceramist and the department psychologist, who supervised the creative activity and observed the emerging relational dynamics. No therapies were administered during the clay therapy.

To evaluate the effect of clay therapy on emotional status of the patients, we performed the VAS and the ArtsObs scale. To evaluate the effect of clay therapy on physical status, we investigated the presence of pain, fatigue and nausea. Measurements were taken before the start of the workshop (T0) and within 15 min after its conclusion (T1). The resulting measurements were compared to determine whether there had been an improvement or worsening. To assess whether the child’s participation in clay therapy had repercussions on the parents’ emotional status, we also performed the ArtsObs test at T0 and T1 on the parents and compared the results.

To analyze the relationship between demographic characteristics of the children and of the parents and the effectiveness of clay therapy, we performed a correlation test between the demographic characteristics and the variation between T0 and T1 of the emotional and physical indicators.

Informed consent was obtained from the parents or the legal guardians of the enrolled patients. The study was carried out following the Helsinki declaration of human rights and was approved by our Departmental Committee (approval number DIPUSVSP-22-09-2555).

### 2.3. VAS

The Visual Analog Scale (VAS) is a simple, time-efficient tool widely used to assess subjective experiences such as pain, mood, or emotional states in both adults and children [34]. The scale consists of a horizontal or vertical line, typically 10 cm in length, with verbal or pictorial anchors at each end: one end representing the absence of the sensation and the other the maximum intensity of the sensation [35].

In pediatric populations, especially hospitalized children, the VAS is particularly suitable because it allows them to communicate feelings that may be difficult to verbalize, such as anxiety, anger, or arousal. Hospitalization is often experienced as stressful and threatening, and children may feel helpless or emotionally overwhelmed.

In this study, the VAS was adapted to measure four specific emotional domains before and after each clay therapy session:

- Mood: overall emotional state, ranging from negative/low mood to positive/high mood.
- Anxiety: level of worry or nervousness experienced at the time of assessment.
- Anger: intensity of feelings of irritability or frustration.
- Arousal: perceived activation or energy level, from calm/relaxed to highly alert or tense.

The scale consisted of a 10 cm horizontal line with three pictorial representations and numeric anchors from 0 to 10: 0 represented the maximum intensity of the negative emotion (e.g., sadness, anger, anxiety, high arousal); 10 represented the opposite, i.e., absence of the negative emotion or optimal positive state; and 5 indicated a moderate or neutral state. Interval markers along the line guided children in their responses. Children were asked to mark the point corresponding to their perceived status at that moment before (T0) and immediately after (T1) the clay therapy session. Sessions took place in the hospital playroom after routine medical checks, under the supervision of trained psychologists.

### 2.4. ArtsObs Scale

The ArtsObs scale is a systematic, flexible observational tool designed to evaluate participant responses across a broad range of arts-based interventions, including hospital-based programs, residential care settings, and community initiatives. It is suitable for participants of all ages, from infancy to older adulthood, and can assess both passive engagement (e.g., observing music, theater, or dance) and active participation in creative or therapeutic programs [36]. ArtsObs allows unobtrusive observation by trained staff, enabling the collection of data on multiple dimensions.

Specifically, the scale measures:

- Mood: positive or negative affective state, including changes in emotional tone during the activity.
- Relaxation: degree of calmness or reduction in tension observed during the session.
- Distraction: level of attention and focus directed toward the activity, indicating engagement and temporary relief from stressors.
- Behavioral engagement: participation, initiative, and interaction with materials or facilitators.
- Social interaction: verbal and non-verbal interactions with peers, caregivers, or staff during the session.

ArtsObs integrates both quantitative measures—such as standardized mood scores and engagement ratings—and qualitative feedback, including descriptive case notes and narrative observations, within a mixed-methods framework. This dual approach allows for a comprehensive assessment of the immediate and broader impact of arts-based interventions. In the context of pediatric oncology, ArtsObs has demonstrated validity and reliability for evaluating the emotional and behavioral responses of hospitalized [33]. The scale is particularly sensitive to detecting changes in mood, relaxation, distraction, and engagement, providing objective evidence of the psycho-emotional benefits of creative therapies during hospitalization. Although art therapy is a relatively recent addition to supportive pediatric oncology care, existing studies suggest that structured creative interventions can enhance children’s well-being, reduce anxiety, and improve emotional regulation [23,24,25,31,32].

ArtObs is also effective in evaluating caregivers because it allows observing their behavior in a natural situation during the child’s artistic and creative activity [33].

### 2.5. Pain, Fatigue and Nausea Evaluation

To date, there are no objective systems for evaluating symptoms such as pain, nausea, malaise, and fatigue. To evaluate the presence of pain, we adopted the Wong-Baker FACES Pain Rating Scale (WBS) [37]. The evaluation of fatigue and nausea was carried out by assigning a score from 0 to 5 according to the Common Terminology Criteria for Adverse Events (CTCAE).

### 2.6. Statistical Analysis

Given the lack of data on the use of effect of clay therapy on emotional and physical status of children with onco-hematological disease, we set the sample size for this pilot study at n = 40 patients. This number intercepts various expected proportions, with a confidence level of 95% and a margin of error ranging from a minimum of 3.08% (for an expected proportion of 1%) to a maximum of 15.5 (for an expected proportion of 50%).

Continuous data are given as mean and standard deviation (SD) and were compared using *t*-test or Mann–Whitney U tests or Wilcoxon signed-rank test, where appropriate. Categorical data are presented as absolute and percentage frequencies and were compared using the Chi-squared test or Fisher Exact Test, where appropriate. The study of the correlations was carried out using Spearmen test. Cohen’s d test was used to analyzed the effect size. All statistical analyses were performed using XLSTAT version 2024.4.2 by Addinsoft. *p* values < 0.05 were considered statistically significant.

## 3. Results

### 3.1. Demographic and Clinical Characteristics of the Patients and Parents Enrolled

We enrolled 40 patients and their parents (one parent for each patient). The mean age of the patients was 10.7 years (SD 5.15), while the mean age of the parents was 41.4 years (SD 6.61). Female were 22 (55%) and male were 18 (45%) of the patients while the group of the parents was composed of 33 mothers (82.5%) and 7 fathers (17.5%).

Patients’ diagnosis were: 15 (37.5%) brain tumor, 9 (22.5%) lymphomas, 5 (12.5%) bone and soft tissue sarcomas, 5 (12.5%) leukemia, 5 (12.5%) renal tumors and 1 (2.5%) hepatoblastoma.

### 3.2. VAS Results

**Figure 1:**
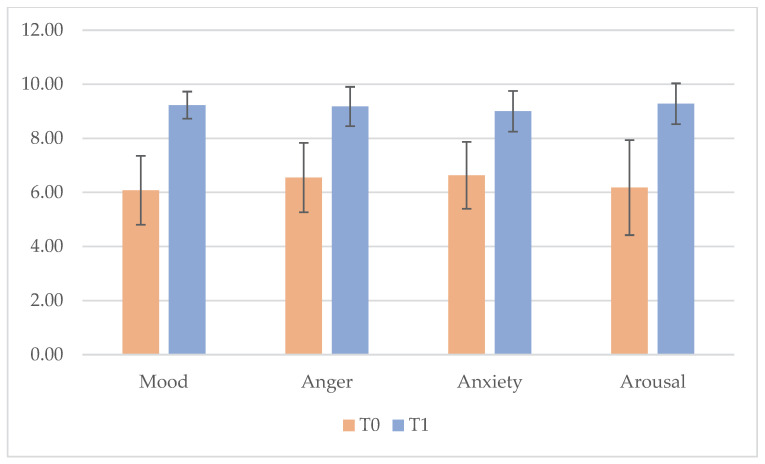
Mean VAS results at T0 and T1. Error bars represent standard deviation.

Before starting the clay therapy, the patients had moderate average value of arousal, anxiety, anger and mood measured through VAS. After clay therapy, each value had a statistically significant increase as showed in Table 1 and Figure 1.

We did not find statistically significant correlations between the variation in VAS results at T0 and T1(Δ T1-T0) with age nor statistically significant difference in Δ T1-T0 VAS scale results according to gender of the patients (Table 2).

### 3.3. ArtsObS

**Figure 2:**
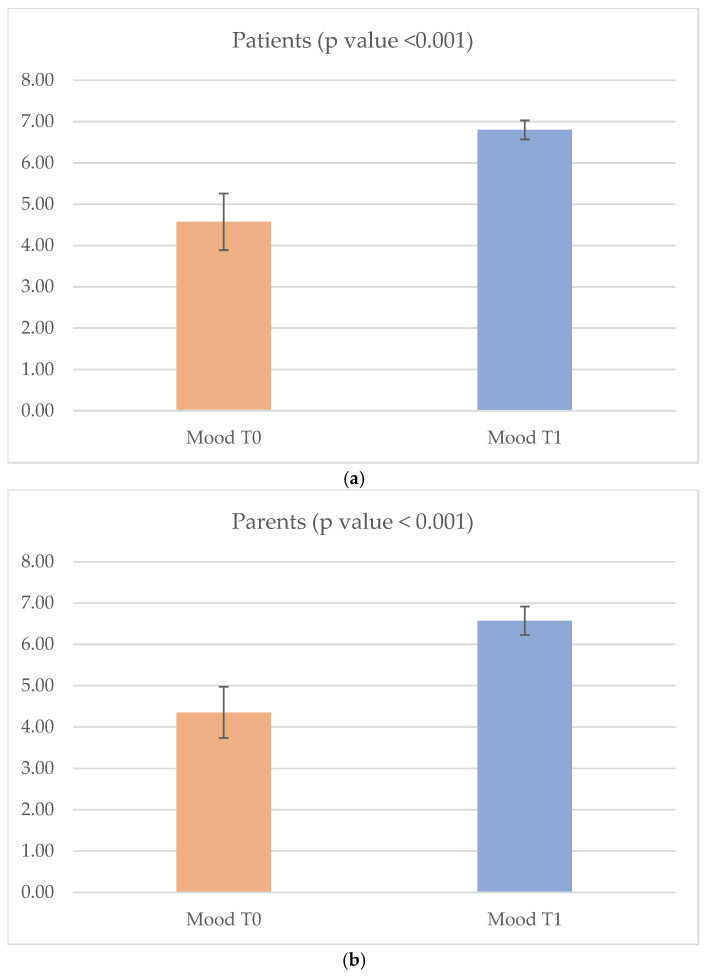
T0 and T1 mood ArtsObs mean value of patients (**a**) and their parents (**b**). Error bars represent standard deviation.

Before starting the clay therapy, patients had a mean value of mood measured through the ArtsObs scale of 4.58 (SD 1.37) with a mean relaxation of 1.95 (SD 0.22; only 2 patients had a relaxation value of 1) and a value of distraction of 2 (SD 0; all patients had a distraction value of 2). At T1 the mood value increased to a mean value of 6.8 (SD 0.46) with a Δ T1-T0 of 2.22 (Figure 2); this increase was statistically significant (*p* value < 0.001). We did not find statistically significant correlations between the variation in mood at T0 and T1 with age (Δ T1-T0) while we observed that female had a statistically significant higher Δ T1-T0 mood results compared to male (*p* < 0.041) (Table 3).

Parents showed a mean mood value of 4.35 at T0 (SD 1.24) and a statistically significant increase to a mean mood value of 6.57 at T1 (SD 0.69) (*p* value < 0.001). Also for parents, we did not find statistically significant relationship between Δ T1-T0 mood and difference according to gender (Table 3).

### 3.4. Pain, Fatigue and Nausea Evaluation Results

**Figure 3:**
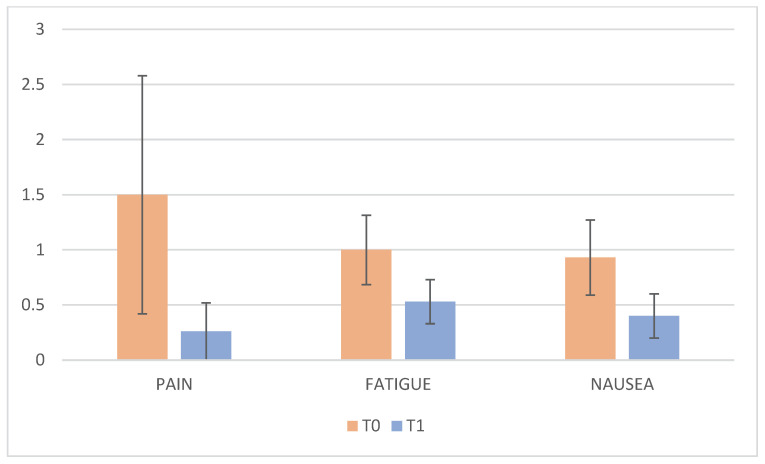
Pain, fatigue and nausea mean values at T0 and T1. Error bars represent standard deviation.

As with emotional parameters, indicators relating to physical state also showed a statistically significant improvement between before and after the clay therapy (Table 4 and Figure 3).

We did not find statistically significant correlations between the variation in symptoms at T0 and T1 (Δ T1-T0) with age nor statistically significant difference in Δ T1-T0 symptoms according to gender of the patients.

## 4. Discussion

A childhood cancer diagnosis represents a profoundly complex and potentially traumatic experience, characterized by invasive treatments, prolonged hospitalizations, and widespread disruptions to daily life, social bonds, and developmental pathways. Evidence consistently indicates that children, adolescents, and young adult cancer survivors are at increased risk of anxiety, depression, and other psychological difficulties compared with their healthy peers [38].

Pediatric oncology therefore involves a complex interplay between biological processes, including disease- and treatment-related effects, and psychological dimensions such as emotional distress, perceived loss of control, functional limitations, and altered social and developmental trajectories. Within this framework, early psychosocial interventions implemented during hospitalization may play a crucial protective role by alleviating emotional suffering and promoting more adaptive coping and adjustment.

The present prospective pilot study provides preliminary evidence that clay therapy has a significant positive impact on the emotional well-being of our sample of hospitalized pediatric oncology patients. Following a single structured clay therapy session, statistically significant improvements were observed across all emotional domains assessed using the Visual Analog Scale, including mood, anxiety, anger, and arousal. These findings indicate that even brief, structured creative interventions could promote rapid emotional regulation and facilitate a shift toward a more positive and relaxed affective state.

Notably, improvements in VAS scores were independent of patients’ age and gender, suggesting that clay therapy may be effective across different developmental stages. This supports the suitability of clay-based interventions as developmentally flexible and inclusive tools within pediatric hospital settings.

Findings obtained using the ArtsObs scale further corroborated these results, showing a significant increase in observed mood levels after the intervention. High and stable levels of distraction and relaxation during the sessions highlight the capacity of clay therapy to foster emotional engagement, attentional absorption, and relief from illness-related distress. These effects are particularly relevant in pediatric oncology, where emotional suffering is often associated with feelings of helplessness, uncertainty, and loss of autonomy related to hospitalization.

Interestingly, female patients demonstrated a significantly greater improvement in observed mood compared with male patients. Although this result should be interpreted cautiously due to the limited sample size, it may reflect gender-related differences in emotional expressiveness or engagement with artistic activities. Further research with larger samples is needed to clarify this finding.

A clinically relevant and novel finding of this study is the indirect emotional benefit observed in parents. Parents exhibited a significant improvement in mood following their child’s participation in clay therapy. This finding underscores the systemic nature of pediatric illness, whereby improvements in a child’s emotional well-being can positively influence caregiver emotional states.

Given that caregivers of children with cancer are at heightened risk of psychological distress, anxiety, and emotional exhaustion, interventions that indirectly support parental well-being represent an important added value. Clay therapy may therefore contribute to family-centered psychosocial care within pediatric oncology settings.

In addition to emotional outcomes, clay therapy was associated with statistically significant reductions in self-reported pain, fatigue, and nausea. Although these symptoms are primarily biologically driven by disease and treatment-related factors, the observed improvements suggest that psychological relaxation and cognitive distraction may modulate symptom perception.

Engagement in clay-based creative activities may reduce stress-related physiological arousal, enhance perceived control, and shift attentional focus away from physical discomfort, thereby contributing to symptom relief. These findings are consistent with existing literature on hospital play and expressive therapies, which demonstrates their effectiveness in reducing distress and symptom burden in hospitalized pediatric populations [30], and are also in line with theoretical frameworks that emphasize the role of expressive and multisensory activities in promoting emotional regulation and coping [28,29]. Clay therapy appears to facilitate active agency and a sense of mastery, allowing children to externalize complex emotional experiences and regain a sense of control within the highly medicalized hospital environment. The effectiveness of clay therapy lies in the specific nature of clay manipulation, as direct contact with the clay stimulates important sensorial, emotional, and expressive components, integrating in a complementary way with other art therapy activities.

This mechanism may be particularly valuable in pediatric oncology, where patients often experience loss of autonomy, unpredictability, and heightened emotional distress.

Taken together, these findings support the integration of clay therapy, into standard supportive care for pediatric oncology patients. Providing structured creative play opportunities during hospitalization addresses a critical developmental need, as illness, stress, and physical limitations often interfere with spontaneous play and social interaction—both essential for healthy emotional and cognitive development. Furthermore, other previous studies have highlighted similar results with other creative activities such as music and painting [39].

By promoting emotional expression, relaxation, and engagement, clay therapy may enhance coping skills, foster psychological resilience, and strengthen the therapeutic alliance between patients, families, and healthcare professionals. Improved emotional regulation may also facilitate treatment adherence and cooperation during medical procedures, contributing to a comprehensive biopsychosocial model of pediatric oncology care.

The observations of our study are limited by the fact that we were unable to conduct a randomized trial at this time (possible biases in our study include the lack of randomization and blinding). Furthermore, our study focused on evaluating the immediate effect of ceramic therapy on the emotional and physical state of patients and parents and based on our data we are unable to evaluate any long-term beneficial effects. We hope the results obtained in this pilot study will justify the design of a larger future study which also includes longitudinal evaluations over time.

## 5. Conclusions

The findings of this pilot study support the feasibility and potential clinical value of clay therapy as a structured, creative intervention in pediatric oncology. Our results indicate that clay therapy is associated with significant improvements in emotional well-being, as reflected in reductions in anxiety, anger, and arousal, and enhancements in positive mood states among our sample of hospitalized children. Moreover, children reported decreased perceptions of pain, fatigue, and nausea, suggesting that clay therapy may also contribute to physical comfort and symptom management, potentially by providing distraction, engagement, and sensory grounding during treatment.

In addition to direct patient benefits, the intervention may have indirect positive effects on caregivers’ emotional well-being, as the children’s engagement and visible emotional regulation can reduce parental stress and anxiety.

Although preliminary, these findings provide empirical support for the potential efficacy of clay therapy in enhancing both emotional and physical aspects of quality of life in pediatric oncology. The study emphasizes that structured creative interventions are not merely recreational but represent therapeutically meaningful activities capable of mitigating the psycho-emotional burden of hospitalization and treatment.

Future research should focus on larger, controlled studies to confirm these effects, explore the mechanisms underlying clay therapy’s benefits, and examine long-term outcomes on resilience, coping strategies, and developmental trajectories.

In conclusion, clay therapy emerges as a low-risk and accessible tool to support the holistic care of children with cancer, enhancing emotional regulation, reducing stress, and fostering a sense of agency during a profoundly challenging period of life. Its incorporation into multidisciplinary care programs can be useful to improve the overall well-being and quality of life of pediatric oncology patients.

## Figures and Tables

**Table 1:** VAS results at T0 and T1.

	T0		T1				Δ T1-T0
VAS	Mean	SD	Mean	SD	*p* Value	d	Mean Δ
Arousal	6.18	3.51	9.28	1.50	<0.0001	1.15	+3.1
Anxiety	6.63	2.48	9.00	1.50	<0.0001	1.3	+2.37
Anger	6.55	2.56	9.18	1.45	<0.0001	1.26	+2.63
Mood	6.08	2.55	9.23	1.00	<0.0001	1.62	+3.15

Δ T1-T0 = variation between T0 and T1; SD = standard deviation; d = Cohen’s d effect size.

**Table 2:** Difference in Δ T1-T0 according to gender.

	Female		Male			
VAS	Mean	SD	Mean	SD	*p* Value	d
Mood	3.455	3.455	2.778	2.602	0.234	0.2
Anger	2.773	1.926	2.444	2.431	0.478	0.15
Anxiety	2.136	2.145	2.667	2.612	0.629	0.22
Arousal	3.591	3.319	2.500	2.854	0.393	0.35

Δ T1-T0 = variation between T0 and T1; SD = standard deviation, d = Cohen’s d effect size.

**Table 3:** Mood Δ T1-T0 stratified according to gender.

	Female Mood Δ T1-T0		Male Mood Δ T1-T0			
Subjects	Mean	SD	Mean	SD	*p* Value	d
Patients	2.500	1.058	1.889	1.183	0.041	0.54
Parents	2.121	1.269	1.714	1.254	0.531	0.32

Δ T1-T0 = variation between T0 and T1; SD = standard deviation, d = Cohen’s d effect size.

**Table 4:** Pain, fatigue and nausea at T0 and T1.

	T0		T1				Δ T1-T0
Symptoms	Mean	SD	Mean	SD	*p* Value	d	Mean Δ
Pain	1.5	2.16	0.26	0.57	0.03	0.78	−1.24
Fatigue	1	0.63	0.53	0.49	0.01	0.83	−0.47
Nausea	0.93	0.68	0.4	0.49	0.01	0.89	−0.53

Δ T1-T0 = variation between T0 and T1; SD = standard deviation, d = Cohen’s d effect size.

## Data Availability

The raw data supporting the conclusions of this article will be made available by the authors on request.

## References

[1] Merks J.H., Ozgen H.M., Koster J., Zwinderman A.H., Caron H.N., Hennekam R.C. (2008). Prevalence and patterns of morphological abnormalities in patients with childhood cancer. JAMA.

[2] Van Cleve L., Bossert E., Beecroft P., Adlard K., Alvarez O., Savedra M.C. (2004). The pain experience of children with leukemia during the first year after diagnosis. Nurs. Res..

[3] Sourkes B.M. (1995). Armfuls of Time: The Psychological Experience of a Child with Life-Limiting Illness.

[4] Perricone G., Morales M.R., Polizzi C., Fontana V. (2010). Schemi narrativi sul sé e autostima nel bambino con neoplasia: Uno studio pilota pre-test. Minerva Pediatr..

[5] Guarino A., Serantoni G., Benini F., Orzalesi M. (2010). Gli stili di coping al dolore in età evolutiva e la loro influenza sull’indice di stress genitoriale: Uno studio sperimentale. Atti del XXIII Congresso Nazionale Della Sezione di Psicologia Dello Sviluppo e dell’Educazione.

[6] Hildenbrand A.K., Clawson K.J., Alderfer M.A., Marsac M.L. (2011). Coping with pediatric cancer: Strategies employed by children and their parents to manage cancer-related stressors during treatment. J. Pediatr. Oncol. Nurs..

[7] Miller K.S., Vannatta K., Compas B.E., Vasey M., McGoron K.D., Salley C.G., Gerhardt C.A. (2009). The role of coping and temperament in the adjustment of children with cancer. J. Pediatr. Psychol..

[8] Moore B.D. (2000). Neurocognitive outcomes in survivors of childhood cancer. J. Pediatr. Psychol..

[9] Patenaude A.F., Kupst M.J. (2005). Psychosocial functioning in pediatric cancer. J. Pediatr. Psychol..

[10] Phipps S., Steele R.G., Hall K., Leigh L. (2001). Repressive adaptation in children with cancer: A replication and extension. Health Psychol..

[11] (2009). SIOP. Atti del XXXXI Congresso Mondiale della Società Internazionale di Oncologia Pediatrica. Pediatr. Blood Cancer.

[12] Baltes P.B., Reese H.W., Lipsitt L.P. (1980). Life-span developmental psychology. Annu. Rev. Psychol..

[13] Perricone Briulotta G. (2005). Manuale di Psicologia dell’Educazione. Una Prospettiva Ecologica per lo Studio e l’Intervento sul Processo Educativo.

[14] Enskär K., von Essen L. (2000). Important aspects of care and assistance for children with cancer. J. Pediatr. Oncol. Nurs..

[15] Canning E.H., Canning R.D., Boice W.T. (1992). Depressive symptoms and adaptive style in children with cancer. J. Am. Acad. Child. Adolesc. Psychiatry.

[16] Allen R., Newman S.P., Souhami R.L. (1997). Anxiety and depression in adolescents with cancer: Findings in patients and parents at the time of diagnosis. Eur. J. Cancer.

[17] Sawyer H. (2000). Meeting the information needs of cancer patients. Prof. Nurse.

[18] Stewart D.E., Cameron J.I., Franche R.L., Cheung A.M. (2002). Lifestyle interference and emotional distress in family caregivers of advanced cancer patients. Cancer.

[19] Beale I.L., Bradlyn A.S., Kato P.M. (2003). Psychoeducational interventions with pediatric cancer patients: Part II. Effects of information and skills training on health-related outcomes. J. Child. Fam. Stud..

[20] Hinds P.S., Burghen E.A., Haase J.E., Phillips C.R. (2006). Advances in defining, conceptualizing, and measuring quality of life in pediatric patients with cancer. Oncol. Nurs. Forum..

[21] Rollins J., Bolig R., Mahan C. (2005). Meeting Children’s Psychosocial Needs Across the Health-Care Continuum.

[22] Favara-Scacco C., Smirne G., Schilirò G., Di Cataldo A. (2001). Art therapy as support for children with leukemia during painful procedures. Med. Pediatr. Oncol..

[23] Madden J.R., Mowry P., Gao D., Cullen P.M., Foreman N.K. (2010). Creative arts therapy improves quality of life for pediatric brain tumor patients receiving outpatient chemotherapy. J. Pediatr. Oncol. Nurs..

[24] Kaimal G., Ray K., Muniz J. (2016). Reduction of cortisol levels and participants’ responses following art making. Art. Ther..

[25] Schore A.N. (2001). Effects of a secure attachment relationship on right brain development, affect regulation, and infant mental health. Infant Ment. Health J..

[26] Kagin S.L., Lusebrink V.B. (1978). The expressive therapies continuum. Art. Psychother..

[27] Malchiodi C.A. (2012). Understanding Children’s Drawings.

[28] Li W.H.C., Chung J.O.K., Ho K.Y., Kwok B.M.C. (2016). Play interventions to reduce anxiety and negative emotions in hospitalized children. BMC Pediatr..

[29] Walker C. (1989). Use of art and play therapy in pediatric oncology. J. Pediatr. Oncol. Nurs..

[30] Gunter M. (2000). Art therapy as an intervention to stabilize the defenses of children undergoing bone marrow transplantation. Arts Psychother..

[31] Paul-Dauphin A., Guillemin F., Virion J.M., Briançon S. (1999). Bias and precision in visual analogue scales: A randomized controlled trial. Am. J. Epidemiol..

[32] Aitken R.C. (1969). Measurement of feelings using visual analogue scales. Proc. R. Soc. Med..

[33] Fancourt D., Poon M. (2016). Validation of the Arts Observational Scale (ArtsObS) for the evaluation of performing arts activities in health care settings. Arts Health.

[34] Avola M., Garibaldi E., La Spina M., Di Cataldo A., Russo G., Lo Nigro L., Montanaro M., Scarponi D., Militello A., Raciti C. (2025). Art therapy and its impact on mood and emotional states in pediatric hematology oncology units: Translation and validation of the Italian version of the Arts Observational Scale (ArtsObS). Healthcare.

[35] Rollins J.A. (2005). Tell me about it: Drawing as a communication tool for children with cancer. J. Pediatr. Oncol. Nurs..

[36] Attinà G., Romano A., Maurizi P., D’Amuri S., Mastrangelo S., Capozza M.A., Triarico S., Ruggiero A. (2021). Management of oral mucositis in children with malignant solid tumors. Front. Oncol..

[37] National Cancer Institute (2017). Common Terminology Criteria for Adverse Events (CTCAE).

[38] Lee A.R.Y.B., Low C.E., Yau C.E., Li J., Ho R., Ho C.S.H. (2023). Lifetime burden of psychological symptoms, disorders, and suicide due to cancer in childhood, adolescent, and young adult years: A systematic review and meta-analysis. JAMA Pediatr..

[39] Giordano F., Muggeo P., Rutigliano C., Barzaghi F., Battisti L., Coccia P., Colombini A., D’Amico M.R., De Santis R., Mascarin M. (2023). Use of music therapy in pediatric oncology: An Italian AIEOP multicentric survey study in the era of COVID-19. Eur. J. Pediatr..

